# Extracellular vesicles in host-parasite interactions: a bibliometric review of mechanisms, diagnostics, vaccines, and drug delivery (2015–2025)

**DOI:** 10.3389/fcimb.2026.1859796

**Published:** 2026-06-04

**Authors:** Xin Wang, Jingyuan Li, Yuan Zhang, Hailong Guo, Di Zhang, Wen Zhao, Zengru Xie

**Affiliations:** 1 Department of Minimally Invasive Spine Surgery and Precision Orthopedics, The First Affiliated Hospital of Xinjiang Medical University, Urumqi, Xinjiang, China; 2 Department of Orthopedics and Trauma, the First Afliated Hospital of Xinjiang Medical University, Urumqi, Xinjiang, China; 3 Xinjiang Engineering Technology Research Center for Medicine-Industry Integration, Xinjiang Medical University, Urumqi, Xinjiang, China; 4 Medical Laboratory Center, The First Affiliated Hospital of Xinjiang Medical University, Urumqi, Xinjiang, China; 5 Key Laboratory of High Incidence Disease Research in Xingjiang (Xinjiang Medical University), Ministry of Education, Urumqi, Xinjiang, China; 6 Xinjiang Clinical Research Center for Orthopedics, Xinjiang Medical University, Urumqi, Xinjiang, China

**Keywords:** bibliometrics, extracellular vesicles, host−parasite interactions, immunomodulation, parasitic infections

## Abstract

Extracellular vesicles (EVs) derived from parasites have emerged as a critical frontier for understanding pathogenic mechanisms and developing novel control strategies. This bibliometric analysis systematically examines 365 original research articles published between 2015 and 2025 using CiteSpace to map the intellectual structure, collaborative networks, and evolving research hotspots of this field. Global publication output exhibits a sustained upward trend, with China, the United States, Brazil, Spain, and Australia as the leading contributors. Notably, Spain demonstrates the highest centrality in international collaboration networks, functioning as a key hub. Author co-authorship analysis reveals a relatively sparse network density, with Ana Claudia Torrecilhas, Alex Loukas, and Antonio Marcilla among the most prolific contributors. Keyword clustering using the log-likelihood ratio algorithm identifies three predominant research domains: protozoan parasites (encompassing *Plasmodium*, *Trypanosoma*, *Leishmania*, and *Toxoplasma*), trematode parasites (including *Schistosoma* and *Fasciola*), and cellular/molecular mechanisms of immunomodulation. Timeline analysis documents a decisive paradigm shift from morphological description and cargo cataloguing toward mechanistic dissection of host–parasite crosstalk, with increasing emphasis on translational applications in diagnostics, vaccine development, and drug delivery. Burst detection analysis reveals sustained citation bursts for methodological standardization guidelines, reflecting the community’s recognition of persistent challenges in EV isolation and characterization. This technology-driven, interdisciplinary field provides robust evidence for future priority setting, international collaboration, and resource allocation.

## 1 Introduction

Parasitic infections, particularly Neglected Tropical Diseases (NTDs), remain a significant and intractable challenge to global public health. Despite decades of coordinated control programs, the burden of diseases such as malaria, schistosomiasis, leishmaniasis, and Chagas disease continues to be disproportionately borne by impoverished populations in low- and middle-income countries, perpetuating health inequity (Wang et al., 2025). The stagnation in reducing this disease burden is largely attributable to critical gaps in diagnostics, therapeutics, and vaccines. Standard microscopic methods lack sufficient sensitivity for detecting low-intensity and early-stage infections, while the therapeutic armamentarium is severely limited and threatened by the emergence of drug-resistant parasite strains (Ertabaklar et al., 2023; Richard and Yusof, 2018). Furthermore, despite intensive research efforts, safe and highly efficacious vaccines against most human parasites remain in developmental stages (You et al., 2023). Underlying these translational roadblocks is a fundamental and systematic lack of in-depth understanding of the complex molecular dialogue that governs parasite–host interactions. This pressing need for mechanistic insight is shared by research teams worldwide, including our own group at the First Affiliated Hospital of Xinjiang Medical University and the State Key Laboratory on Pathogenesis, Prevention, and Treatment of High Incidence Diseases in Central Asia, which has long been dedicated to parasitology research.

Over the past decade, extracellular vesicles (EVs)—nanoparticles actively released by cells and composed of a lipid bilayer enclosing a diverse cargo of proteins, lipids, and nucleic acids—have emerged as a paradigm-shifting concept in molecular parasitology. Originally considered cellular debris, these vesicles are now recognized as key mediators of intercellular and cross-kingdom communication. Parasite-derived EVs have been demonstrated to transport virulence factors, modulate host immune responses, and facilitate tissue invasion and colonization (You et al., 2023). The functional repertoire of EVs is largely determined by their cargo, which includes a wide range of small regulatory RNAs, such as microRNAs (miRNAs) and tRNA-derived fragments, capable of altering host gene expression in a targeted manner (Yang et al., 2024). Crucially, the unique biological properties of EVs present a dual and transformative opportunity for parasitology from a translational perspective. First, their specific molecular signatures offer new pathways for the development of highly sensitive and non-invasive diagnostic biomarkers. Second, their natural capacity to carry parasite antigens and to be engineered as delivery vehicles positions them as an ideal platform for next-generation vaccine development and targeted drug delivery against parasitic diseases (Barnadas-Carceller et al., 2024; Borgheti-Cardoso et al., 2020; Vidal et al., 2025).

Consequently, understanding the biogenesis, cargo sorting, and downstream functional impacts of EVs in the context of host–parasite interactions has become a cornerstone for bridging fundamental parasitology with clinical innovation. The rapid expansion of this field, however, has resulted in a highly fragmented research landscape. While individual studies have elucidated the roles of EVs from specific parasites—ranging from the delivery of immunomodulatory miRNAs by *Schistosoma japonicum* EVs to the transfer of drug-resistance genes through *Leishmania* EVs—a comprehensive, data-driven synthesis of the field’s evolution, collaborative networks, and knowledge structure is lacking.

To address this gap, the present study employs CiteSpace, a well-established bibliometric software, to conduct a systematic visual analysis of the literature on parasite-derived EVs published between 2015 and 2025. By integrating quantitative metrics—including country/institution/author co-occurrence networks, keyword clustering, and burst detection analysis of both publications and citations—this study aims to objectively delineate the global distribution of research forces, identify predominant thematic clusters, and uncover the temporal dynamics of emerging research frontiers. Unlike a traditional narrative review, this bibliometric approach provides an evidence-based, macroscopic view of the field, intended to inform future priority setting, facilitate international collaboration, and optimize resource allocation in this technologically driven and translationally promising domain of parasitology.

## 2 Data and methods

### 2.1 Data acquisition

A comprehensive literature search was conducted on May 10, 2026, across three databases: the Web of Science Core Collection (Science Citation Index Expanded, SCI-Expanded), PubMed, and the Cochrane Library. The search covered the period from January 1, 2015, to December 31, 2025. The complete search strategy employed in the Web of Science Core Collection is provided below; analogous strategies were adapted for PubMed and the Cochrane Library to ensure consistent and rigorous retrieval across all data sources.

The search was performed using a title (TI) field query combining terms for parasites and extracellular vesicles. The complete search string was as follows: TI = (parasite OR “parasitic disease*” OR malaria OR plasmodium OR leishman* OR trypanosom* OR toxoplasm* OR babesia OR theileria OR amoeb* OR entamoeba OR naegleria OR acanthamoeba OR giardia* OR cryptosporid* OR trichomonas OR blastocystis OR microsporid* OR isospor* OR cyclospor* OR sarcocystis OR helminth* OR nematode* OR cestode* OR trematode* OR schistosom* OR bilharzia OR filaria* OR “lymphatic filariasis” OR elephantiasis OR ascari* OR trichuris OR whipworm OR strongyloides OR hookworm OR ancylostoma OR necator OR dracunculus OR anisakis OR angiostrongylus OR echinococc* OR hydatid* OR hymenolepis OR taenia OR cysticerc* OR clonorchis OR opisthorchis OR fasciola OR paragonim* OR trichinella* OR capillaria* OR gnathostoma* OR thelazia* OR dirofilaria* OR wuchereria* OR brugia* OR onchocerca* OR diphyllobothrium* OR spirometra* OR dipylidium* OR fasciolopsis* OR echinostoma* OR heterophyes* OR metagonimus* OR balantidium* OR toxocara OR baylisascaris OR enterobius OR pinworm OR pentastom* OR acanthocephal*) AND (exosome* OR microvesicle* OR “extracellular vesicle*” OR EV OR EVs OR ectosome* OR “apoptotic bod*”).

### 2.2 Inclusion and exclusion criteria

**Figure 1:**
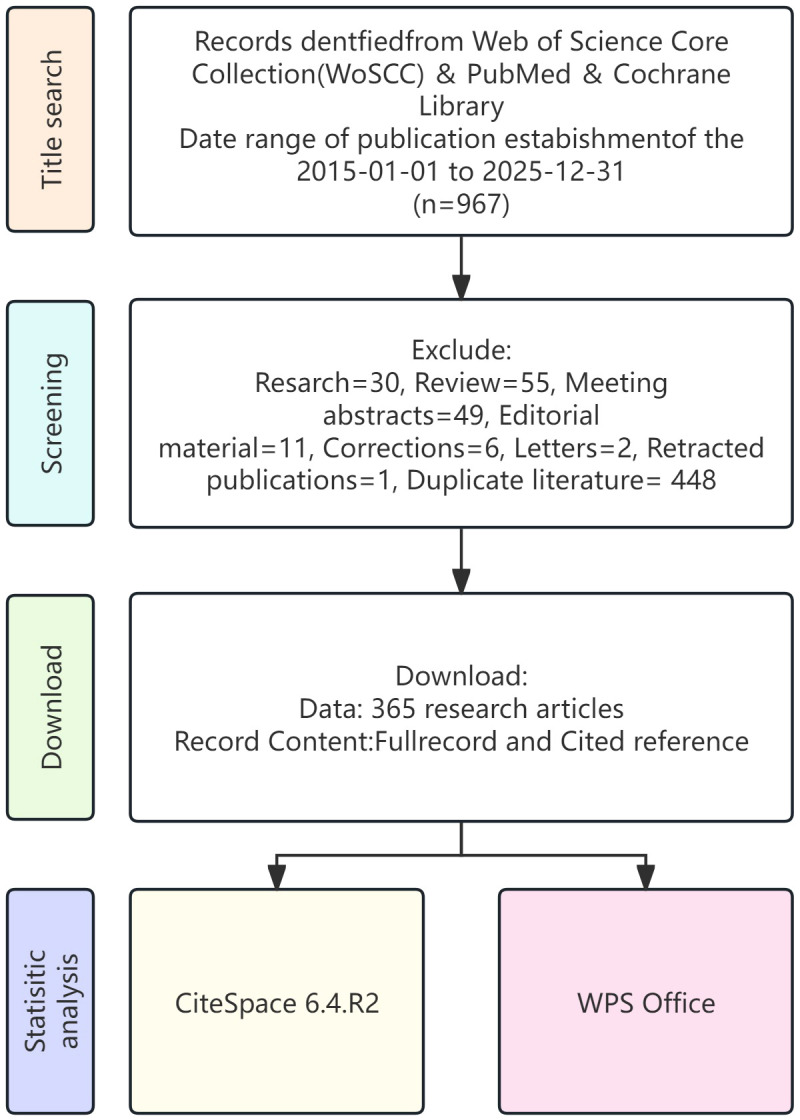
Flow diagram of the literature screening and selection process.

The initial search across the three databases yielded a total of 967 records. During the subsequent screening stage, we systematically excluded the following non-original or secondary literature types: REVIEW, MEETING ABSTRACT, EDITORIAL MATERIAL, CORRECTION, LETTER, and RETRACTED PUBLICATION. This step ensured that only peer-reviewed original research articles, representing the most direct and primary research outputs of the field, were retained for analysis. Duplicate records across databases were identified and removed. Following this screening and deduplication process, a final set of 365 eligible records was retained (**Figure 1**). Full bibliometric data, including cited references and abstracts, were exported in plain text format to ensure full compatibility with CiteSpace software.

### 2.3 Analytical framework

Literature visualization and bibliometric analysis were conducted using CiteSpace (version 6.4.R2). After the exported data were automatically converted into a standardized format upon import, software parameters were set as follows: the time span was defined from 2015 to 2025 with a one-year time slice; the node type was set to “keyword” for thematic analyses; the selection criteria employed the g-index algorithm with a scale factor k = 25 to balance network size and quality; and the network was pruned using the Pathfinder algorithm to highlight its core structure. All other parameters were kept at their default settings.

Keyword co-occurrence networks were constructed and subsequently clustered using the log-likelihood ratio (LLR) algorithm to identify major thematic domains. Clustering was performed in an unsupervised manner, and all clusters were generated automatically based solely on keyword co−occurrence relationships, without any predefined classification system. The modularity (Q) value and weighted mean silhouette (S) score were calculated to evaluate the significance and robustness of the resulting community structure. A Q value greater than 0.3 indicates a significant community structure, while an S score above 0.7 reflects reliable and convincing clustering results (Wang et al., 2025; Wang et al., 2023). In addition, dual-map overlay analysis was performed to visualize interdisciplinarity at the journal level, and burst detection was applied to both keywords and cited references to identify transient research hotspots and milestone publications with sudden surges in attention over time.

## 3 Results

### 3.1 Annual growth trend

**Figure 2:**
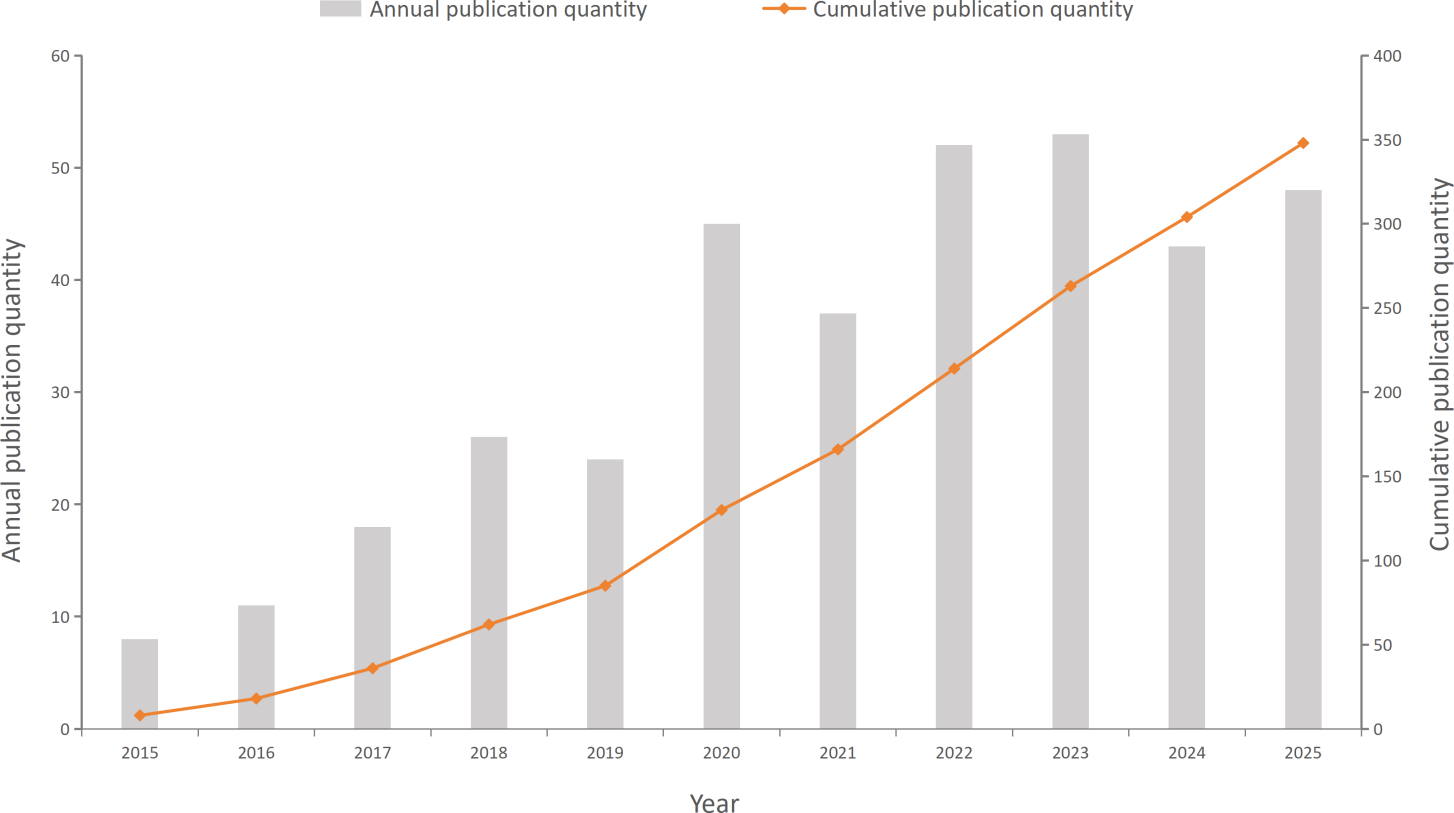
Annual and cumulative publication trends of parasite-derived EV research (2015–2025).

Over the past decade, the global field of parasite-derived extracellular vesicles has exhibited an overall fluctuating upward trend in publication output (**Figure 2**). From 2015 to 2020, the number of published articles experienced steady and significant growth, reflecting an expanding community of researchers entering this domain. Although a slight decline was observed in 2021, the volume of publications rebounded and stabilized at a high level thereafter, peaking in 2023. The sustained strong publication output in the most recent years indicates not only a mature scale of research activity in this field, but also that numerous unresolved scientific questions persist, collectively suggesting a robust and promising trajectory for future investigation.

### 3.2 National, institutional, and author collaborative networks

**Figure 3:**
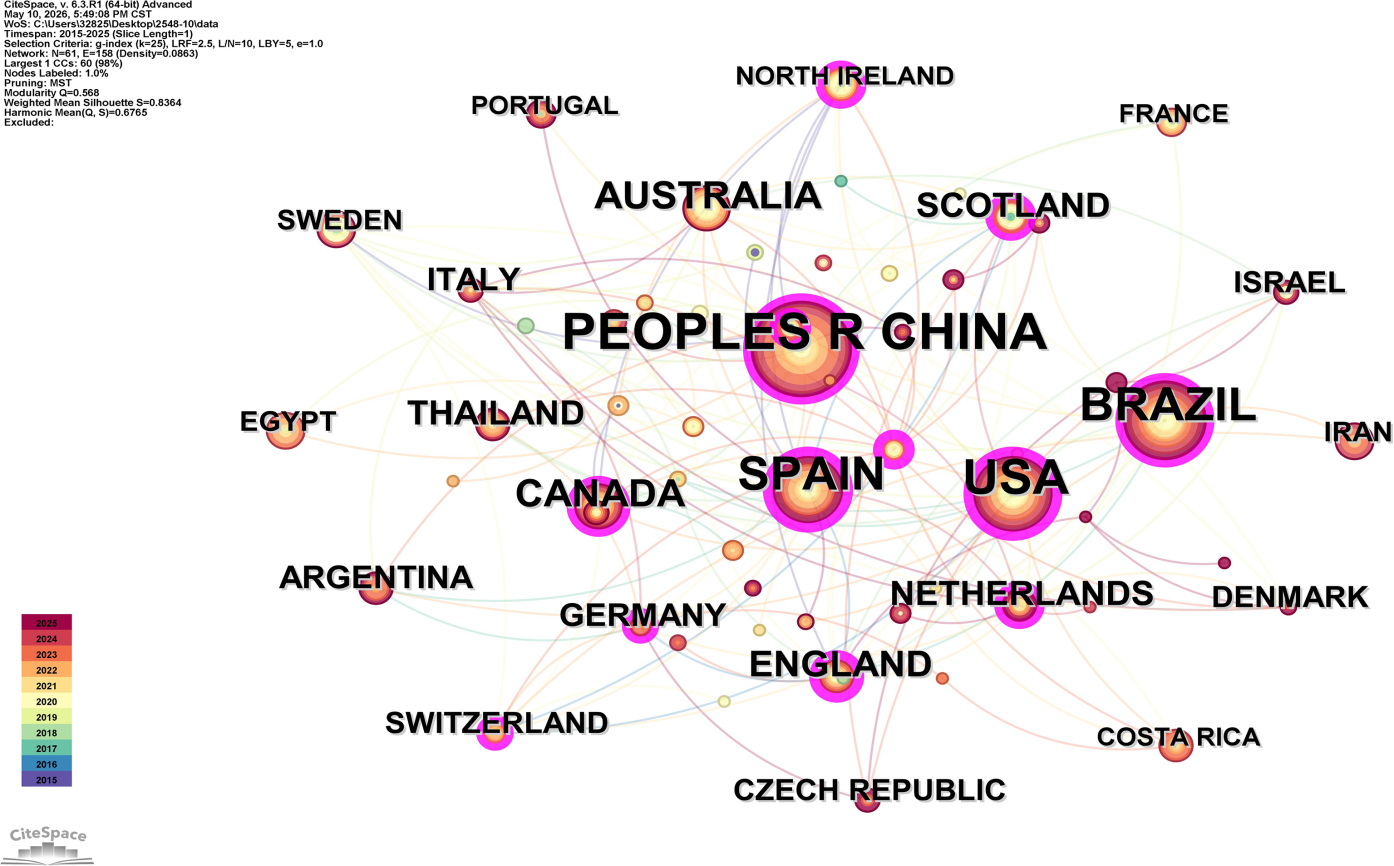
Country co-occurrence network. Node size reflects publication volume; edge thickness indicates collaboration strength.

The CiteSpace-generated country co−occurrence map, illustrated in **Figure 3**, comprises 61 nodes connected by 158 links, indicating that research on parasite−derived EVs has formed a relatively dense network of international knowledge exchange. As detailed in **Table 1**, China (81 publications), the United States (62 publications), Brazil (59 publications), Spain (52 publications), and Australia (27 publications) are the five countries with the highest publication output. However, centrality analysis reveals a contrasting pattern of network influence. Spain (0.30), England (0.27), the United States (0.23), the People’s Republic of China (0.20), and Scotland (0.17) occupy the top five positions in centrality. This contrast underscores that while China and the United States lead in absolute publication volume, their roles as “bridges” in the international collaboration network are relatively less pronounced compared to certain European countries such as Spain and England, which demonstrate both high output and strong network connectivity.

**Table 1:** Top 10 countries/regions contributing to parasite-derived EV research (2015–2025).

Rank	Publication volume	Centrality	Publication year	Country
1	81	0.19	2015	PEOPLES R CHINA
2	62	0.25	2015	USA
3	59	0.12	2017	BRAZIL
4	52	0.30	2015	SPAIN
5	27	0.08	2016	AUSTRALIA
6	26	0.17	2015	CANADA
7	21	0.28	2015	ENGLAND
8	16	0.04	2018	THAILAND
9	15	0.16	2016	SCOTLAND
10	15	0.16	2019	NETHERLANDS

**Figure 4:**
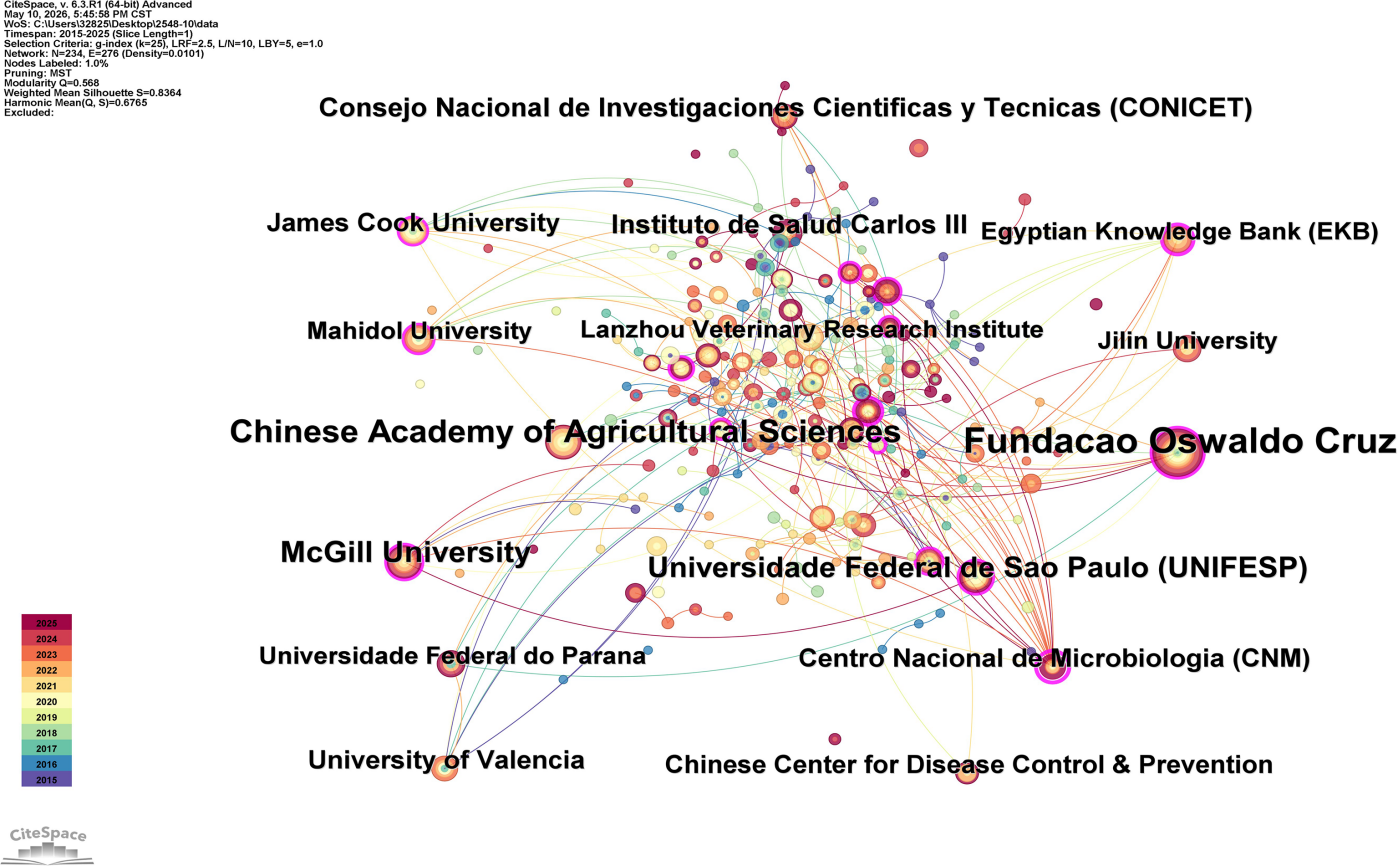
Institutional collaboration network. Node color represents centrality score.

The top ten institutions ranked by publication count are presented in **Table 2**. Among them, Fundacao Oswaldo Cruz (32 publications), the Chinese Academy of Agricultural Sciences (21 publications), McGill University (16 publications), Universidade Federal de Sao Paulo (UNIFESP; 15 publications), and James Cook University (11 publications) rank as the top five in terms of scholarly output. As shown in **Figure 4**, the five institutions with the highest centrality are Centro Nacional de Microbiologia (CNM; 0.68), Aarhus University (0.36), Egyptian Knowledge Bank (EKB; 0.28), the Russian Academy of Sciences (0.28), and Mahidol University (0.20). It is worth noting that CNM exhibits a substantially higher centrality value than all other institutions, highlighting its uniquely influential role as a central hub in the global collaboration network.

**Table 2:** Top 10 institutions contributing to parasite-derived EV research (2015–2025).

Rank	Publication volume	Centrality	Publication year	Institution
1	32	0.17	2017	Fundacao Oswaldo Cruz
2	21	0.06	2016	Chinese Academy of Agricultural Sciences
3	16	0.19	2015	McGill University
4	15	0.11	2018	Universidade Federal de Sao Paulo (UNIFESP)
5	13	0.20	2016	James Cook University
6	12	0.05	2020	Instituto de Salud Carlos III
7	12	0.03	2017	Consejo Nacional de Investigaciones Cientificas y Tecnicas (CONICET)
8	11	0.68	2020	Centro Nacional de Microbiologia (CNM)
9	11	0.09	2015	University of Valencia
10	11	0.18	2016	James Cook University

**Figure 5:**
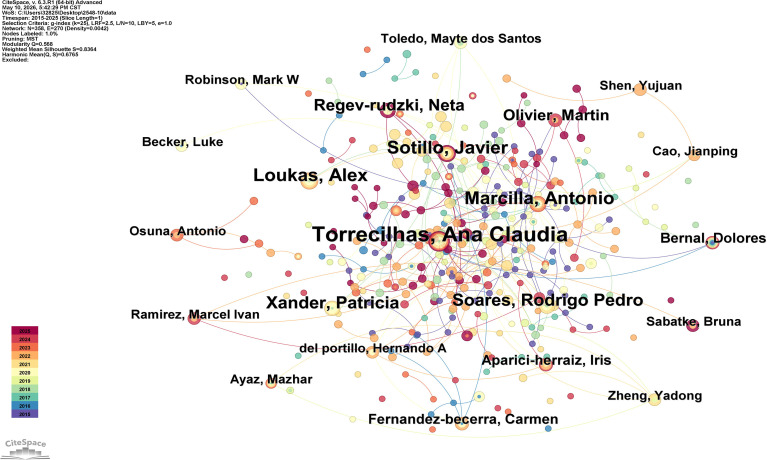
Author co-authorship network (N = 358, E = 270, density=0.0042).

The author co−authorship network, shown in **Figure 5** and summarized in **Table 3**, comprises 358 nodes and 270 links, with a network density of 0.0042. The sparse density indicates that large−scale, densely interconnected research consortia have yet to form, with most collaborations remaining localized within specific research groups. Among the 358 scholars, Ana Claudia Torrecilhas (Federal University of Sao Paulo, Brazil) is the most prolific author with 14 publications, followed by Alex Loukas (James Cook University, Australia) with 9 publications. Antonio Marcilla (University of Valencia, Spain) and Javier Sotillo (Spanish National Centre for Microbiology) have each published 9 papers, tying for second place. Rodrigo Pedro Soares (Fundacao Oswaldo Cruz, Brazil) and Patricia Xander (Federal University of Sao Paulo, Brazil) have each contributed 8 publications.

**Table 3:** Top 8 most prolific authors in parasite-derived EV research (2015–2025).

Author	Publication volume	Country	Institution
Torrecilhas, Ana Claudia	14	Brazil	Universidade Federal de São Paulo (UNIFESP)
Loukas, Alex	9	Australia	James Cook University
Marcilla, Antonio	9	Spain	Universidad de Valencia
Sotillo, Javier	9	Spain	Instituto de Salud Carlos III
Soares, Rodrigo Pedro	8	Brazil	Fundação Oswaldo Cruz (Fiocruz)
Xander, Patricia	8	Brazil	Universidade Federal de São Paulo (UNIFESP)
Regev-rudzki, Neta	7	Israel	Weizmann Institute of Science
Olivier, Martin	6	Canada	McGill University

### 3.3 Keyword analysis

**Figure 6:**
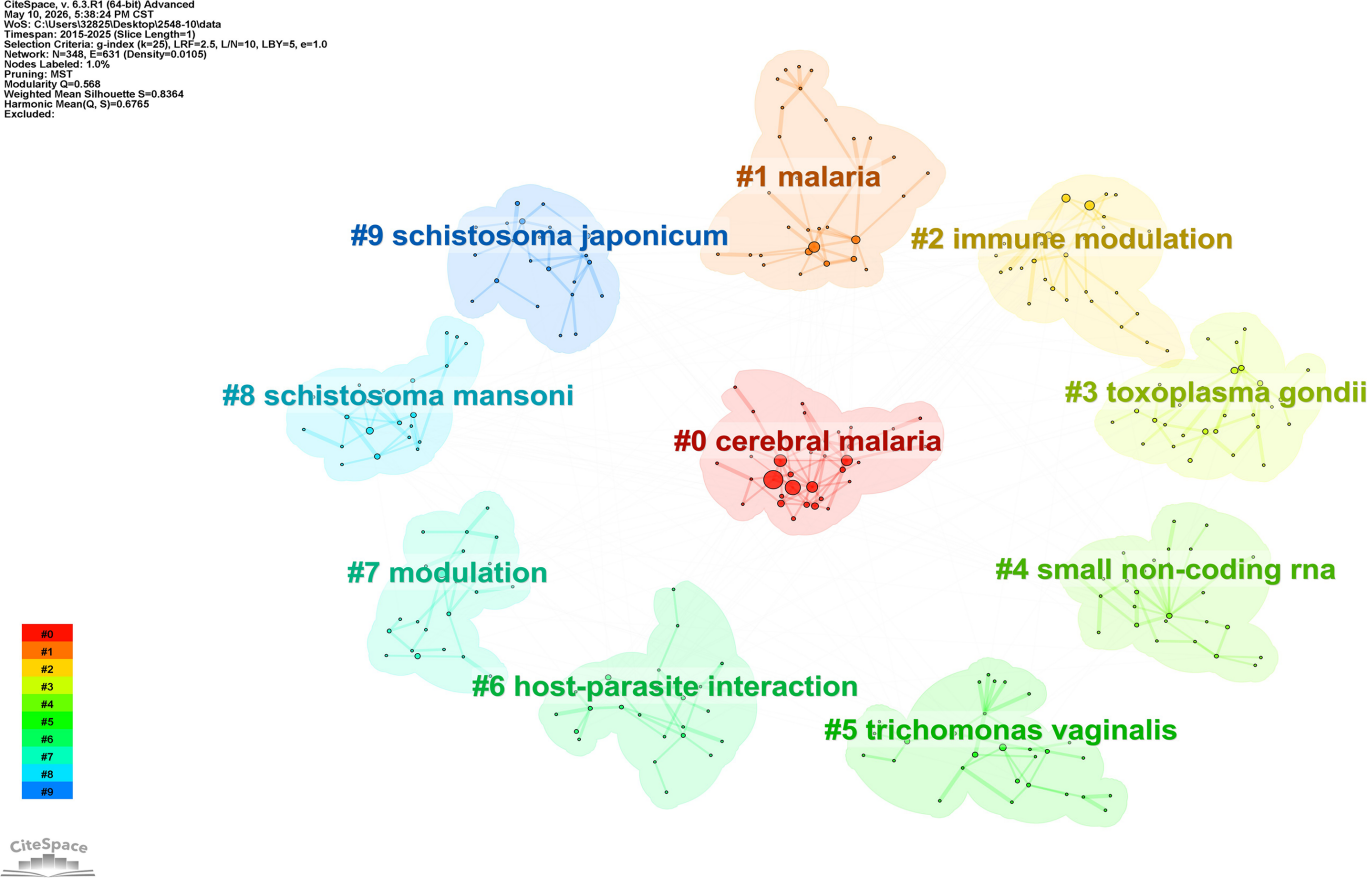
Keyword clustering map (Q = 0.568, S = 0.8364). 11 clusters labeled #0–#9.

Keyword clustering analysis, performed using the LLR algorithm on the co-occurrence network, generated 11 distinct clusters labeled #0 through #10 (**Figure 6**). The network comprised 348 nodes and 631 links, with a density of 0.0105. The modularity value (Q = 0.568) indicates a highly significant community structure, and the weighted mean silhouette score (S = 0.836) reflects efficient and convincing clustering. As shown in **Table 4**, all clusters had silhouette coefficients exceeding 0.7, with values ranging from 0.752 to 0.882, further confirming the high reliability of the cluster assignments. These 11 clusters can be broadly organized into three predominant research domains. The first domain centers on protozoan parasites, prominently featuring cerebral malaria (*Plasmodium* spp., cluster #0), malaria pathogenesis (cluster #1), *Toxoplasma gondii* (cluster #3), and small non-coding RNA-mediated mechanisms (cluster #4). The second domain encompasses trematode parasites, specifically focusing on *Schistosoma mansoni* (cluster #8) and *Schistosoma japonicum* (cluster #9). The third and largest domain is defined by shared cellular and immunological processes, including immune modulation (cluster #2), modulation of host responses (cluster #7), host-parasite interaction (cluster #6), and *Trichomonas vaginalis*-associated mechanisms (cluster #5).

**Table 4:** Summary of keyword clusters identified by LLR (log-likelihood ratio) algorithm.

Cluster ID	Size	Silhouette	Mean (year)	Label (LLR)
0	36	0.786	2018	cerebral malaria; innate immunity; mansoni; leishmania infantum; tgf beta
1	31	0.873	2018	malaria; plasmodium falciparum; cerebral malaria; microparticles; spliced leader rna
2	30	0.814	2021	immune modulation; extracellular vesicles (evs); cryptosporidium; evs characterization; pathogenicity mechanisms
3	29	0.841	2021	toxoplasma gondii; host-pathogen interaction; immunization; murine model; endothelial dysfunction
4	28	0.879	2020	small non-coding rna; trypanosoma cruzi; chagas disease; lipid bodies; small evs
5	28	0.882	2019	trichomonas vaginalis; liver fluke; resistance; biochemical characterization; msrna
6	25	0.876	2020	host-parasite interaction; cutaneous leishmaniasis; responses; nk cells; damps
7	23	0.752	2018	modulation; mechanisms; cystic echinococcosis; acanthamoeba; granulosus infection
8	23	0.784	2018	schistosoma mansoni; naegleria fowleri; vaccine; proteomic analysis; intercellular interactions
9	22	0.776	2021	schistosoma japonicum; schistosomiasis; c-type lectin receptors; rassf1c oncogene; rock pathway

**Figure 7:**
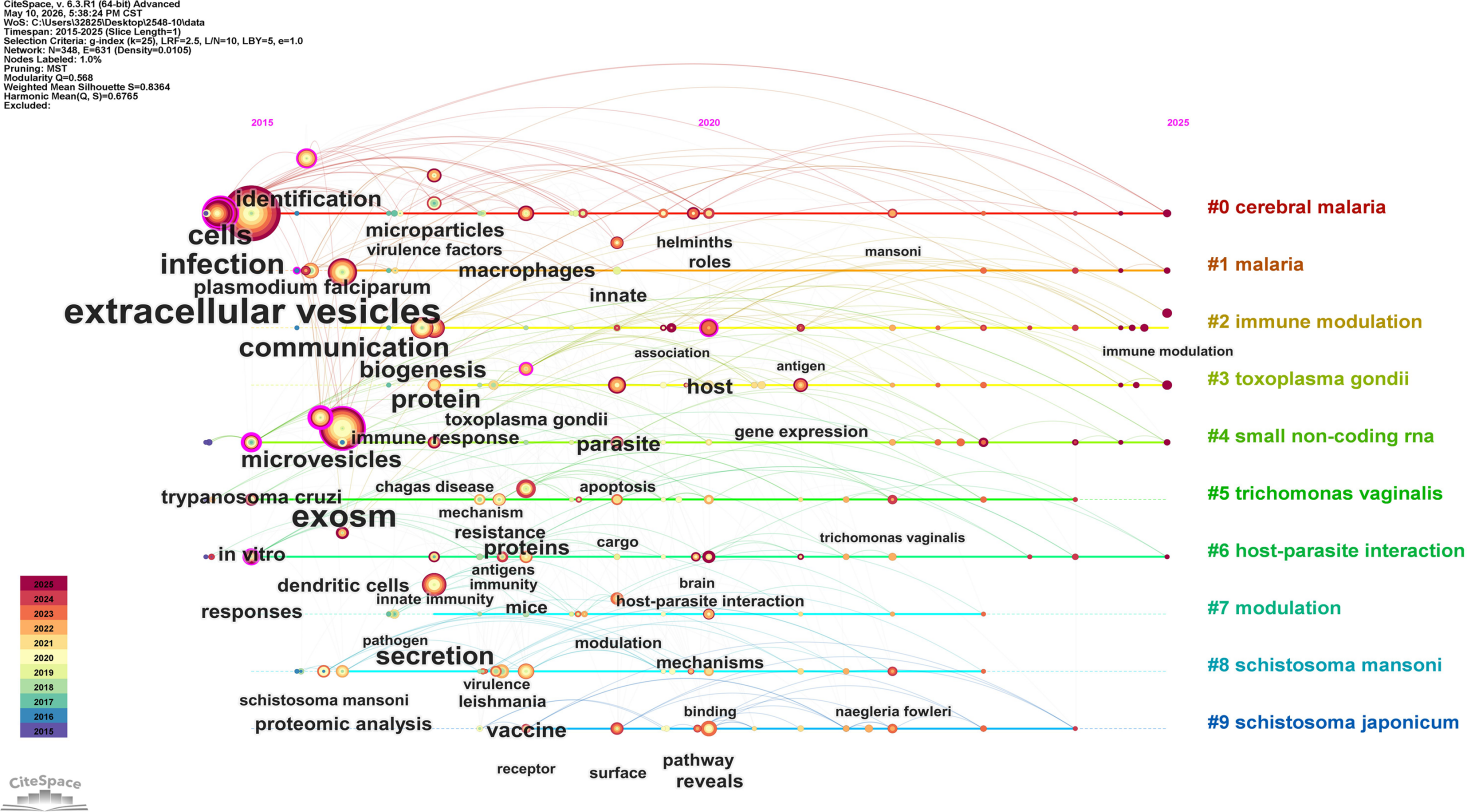
Timeline view of keyword clusters showing temporal evolution of research hotspots.

The timeline view (**Figure 7**) illustrates the evolutionary trajectory of research hotspots across the decade. Early studies (circa 2015–2017) were primarily concentrated on highly pathogenic parasites such as *Plasmodium falciparum*, the foundational biology of EVs, and initial descriptions of their cargo, including small non-coding RNAs. In the mid-period (2018–2020), the research focus shifted toward more detailed molecular mechanisms of immunomodulation, host–parasite crosstalk, and the roles of EVs in malaria pathogenesis and drug delivery. In the most recent years (2021–2025), the field has expanded both in scope and depth. An increasing diversity of parasite taxa—including *Trichomonas vaginalis*, *Entamoeba histolytica*, *Naegleria fowleri*, and various helminths—has appeared as emerging models. Furthermore, research has moved from descriptive cargo profiling toward functional validation of specific EV-derived molecules, particularly miRNAs, in mediating cross-species gene regulation and in shaping the tumor microenvironment in parasite-associated cancers.

### 3.4 Burst citation analysis

**Figure 8:**
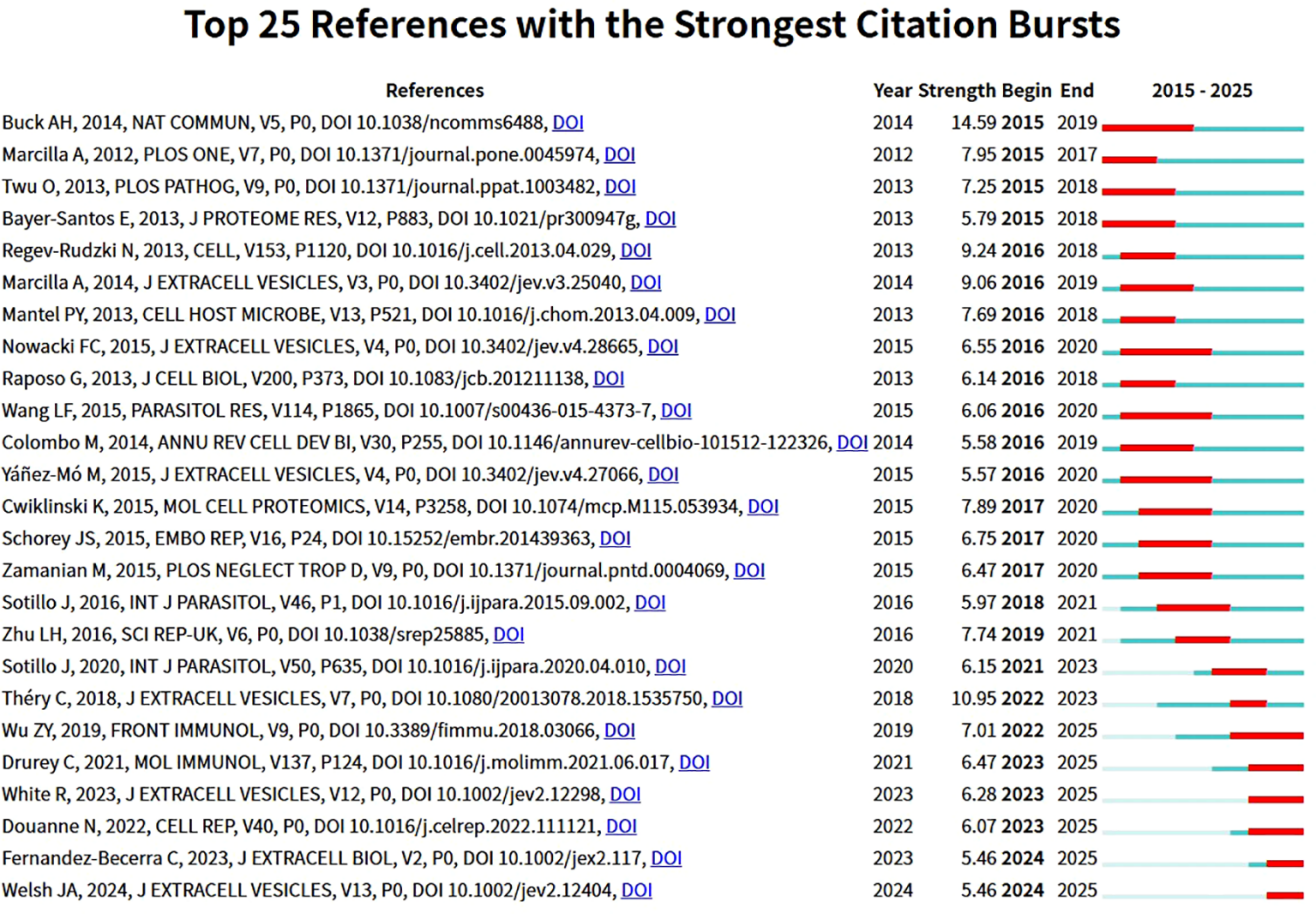
Top 25 references with the strongest citation bursts.

Burst detection analysis of cited references reveals the milestone publications that have experienced the most intense and sudden surges in scholarly attention over the past decade. **Figure 8** lists the top 25 references with the strongest citation bursts. The two most prominent bursts were recorded for Buck et al. (2014) (Buck et al., 2014), a foundational study published in *Nature Communications* that first demonstrated that a murine gastrointestinal parasitic nematode secretes exosomes containing miRNAs and Y RNAs to suppress the host’s type 2 innate immune response (burst strength = 14.59, burst duration: 2015–2019), and Théry et al. (2018) (Théry et al., 2018), which presented the Minimal Information for Studies of Extracellular Vesicles (MISEV2018) guidelines in the *Journal of Extracellular Vesicles* and provided the essential methodological framework that underpinned subsequent EV research (burst strength = 10.95, burst duration: 2022–2023).

Notably, among the 25 references with the strongest citation bursts, seven studies exhibit burst durations that extend actively into 2025. These ongoing bursts encompass studies on *Leishmania* exosome-mediated drug-resistance gene transfer (Douanne et al., 2022), helminth EV interactions with the host immune system (Drurey and Maizels, 2021), community-led guidelines for helminth EV research (White et al., 2023), parasite EV purification guidelines (Fernandez-Becerra et al., 2023), and the MISEV2023 update (Welsh et al., 2024). The persistence of these citation bursts into the most recent analysis window underscores that these works are defining the current frontiers of the field and are likely to continue shaping research trajectories in the immediate future.

### 3.5 Interdisciplinary citation dynamics

**Figure 9:**
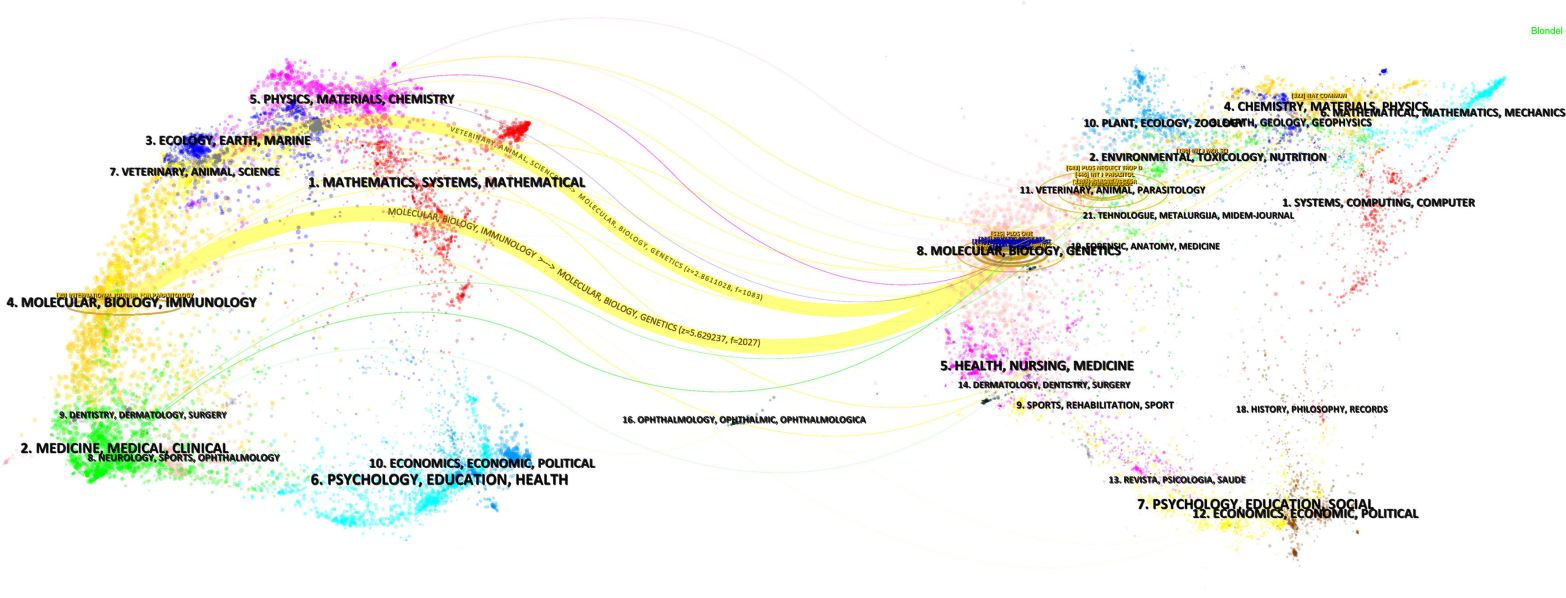
Dual-map overlay of citing and cited journals.

The dual-map overlay analysis, a function of CiteSpace that visualizes the flow of citations between disciplines at the journal level, reveals the interdisciplinary architecture of the parasite-derived EV research field. **Figure 9** illustrates the citation relationships between citing journals (left side) and cited journals (right side). The analysis identifies two dominant citation pathways, represented by thicker colored lines. The primary pathway demonstrates that articles published in journals categorized under MOLECULAR, BIOLOGY, IMMUNOLOGY, and VETERINARY, ANIMAL, SCIENCE are predominantly cited by articles in journals within the MOLECULAR, BIOLOGY, GENETICS disciplinary domain. This pattern underscores the fundamentally interdisciplinary nature of this research domain, wherein foundational knowledge generated in immunology, parasitology, and veterinary science is integrated and advanced through the conceptual and methodological lens of molecular biology and genetics.

## 4 Discussion

### 4.1 Global research landscape and collaborative dynamics

This bibliometric analysis systematically maps a decade of research on parasite-derived extracellular vesicles (EVs), revealing a field that has matured from its foundational descriptive phase into a dynamic, mechanistically oriented, and translationally promising domain. The sustained upward trajectory in publication output, particularly the peak observed in 2023, coincides with several converging developments, including the increasing accessibility of high-throughput technologies for EV cargo profiling and the emergence of translational research programs.

The collaborative network analysis highlights a notable asymmetry in global scientific productivity and influence. China, the United States, and Brazil lead in absolute publication volume; however, the centrality analysis indicates that European nations—most prominently Spain, England, and Scotland—function as the principal hubs of international collaboration. This divergence between productivity and network centrality is reflected in the specific research contributions characteristic of different regions. Research groups in Brazil, led by Torrecilhas and colleagues, have systematically characterized the EVs released by *Trypanosoma cruzi* and *Leishmania* spp., demonstrating that EVs from different *T. cruzi* strains exhibit distinct proteomic profiles and differential effects on host cell infectivity (Ribeiro et al., 2018), and that *Leishmania amazonensis*-derived EVs differentially modulate macrophage and B-1 cell responses in a manner that promotes disease progression (Barbosa et al., 2018). In Spain, Marcilla and colleagues have conducted foundational studies on helminth EVs, establishing that *Fasciola hepatica* EVs from eggs, juveniles, and adults display stage-specific morphologies and proteomes (Sánchez-López et al., 2020), and that EVs from *F. hepatica* and *Dicrocoelium dendriticum* elicit functionally divergent responses in human macrophages, with the former driving a mixed M1/M2 anti-inflammatory phenotype while the latter exerting pro-inflammatory effects (Sánchez-López et al., 2023). These region-specific research programs illustrate how collaborative networks are shaped by both institutional expertise and local epidemiological priorities.

Furthermore, the relatively sparse author co-authorship network (density = 0.0043) indicates that large, interdisciplinary consortia have yet to coalesce in this field. This structural feature represents both a challenge and an opportunity: the formation of broader collaborative networks would likely accelerate progress in key areas, including the development and validation of EV-based biomarkers for neglected parasitic diseases and the standardization of isolation and characterization protocols applicable across diverse parasite taxa and life stages.

### 4.2 Thematic evolution of research hotspots

The keyword clustering and timeline analyses collectively document a field undergoing a decisive transition from morphological and proteomic characterization toward functional and mechanistic interrogation. The early literature was predominantly concerned with establishing that parasites across multiple phyla secrete EVs and with cataloguing their principal protein and nucleic acid cargoes. A landmark study from this initial phase was the work of Szempruch et al. (2016), who demonstrated that *Trypanosoma brucei* EVs function as transferable repositories of virulence factors, mediating the horizontal transfer of serum resistance-associated protein and directly inducing host erythrocyte remodeling and anemia (Szempruch et al., 2016).

Regarding the keyword clustering structure (**Figure 6**), trematodes—particularly schistosomes—formed independent thematic clusters (#8 *Schistosoma mansoni*, #9 *Schistosoma japonicum*), whereas cestodes and nematodes did not form separate clusters. This difference primarily reflects the large volume and thematic concentration of EV research on schistosomiasis (covering isolation, proteomics, immune modulation, and vaccine development), whereas studies on other helminths are more dispersed and their keywords naturally merged into broader clusters such as immune modulation or host-parasite interaction.

Subsequently, the field turned toward elucidating the specific functional consequences of EV-mediated communication during infection. In the context of malaria, Mantel et al. (2016) demonstrated that EVs derived from *Plasmodium falciparum*-infected red blood cells contain functional miRNA-Argonaute 2 complexes that are internalized by endothelial cells, where they modulate target gene expression and alter vascular barrier properties, thus providing a mechanistic framework linking parasite-derived EVs to vascular dysfunction during cerebral malaria (Mantel et al., 2016). In parallel, research on schistosome EVs illuminated how parasite miRNAs manipulate host immune cells. Liu et al. (2019) showed that *Schistosoma japonicum* EV-delivered miR-125b and bantam miRNAs are taken up by host macrophages, increasing their proliferation and TNF-α production in a manner that facilitates parasite survival (Liu et al., 2019).

In the most recent period (2021–2025), the research agenda has expanded in both scope and mechanistic depth. A particularly significant advance has been the functional dissection of individual EV cargo molecules using gain- and loss-of-function approaches. Zhong et al. (2024) demonstrated that *S. japonicum* worm-derived EVs deliver sja-let-7 into hepatic stellate cells, targeting collagen type I alpha 2 chain and downregulating the TGF-β/Smad signaling pathway to attenuate liver fibrosis (Zhong et al., 2024). In a complementary study, Wang et al. (2020) showed that *S. japonicum* egg-derived EVs deliver Sja-miR-71a, which suppresses liver fibrosis by targeting semaphorin 4D and inhibiting TGF-β1/SMAD and IL-13/STAT6 pathways (Wang et al., 2020). These findings collectively reveal a therapeutic dimension to schistosome EV biology that extends beyond their conventional characterization as pathogenic effectors. In a related but mechanistically distinct context, Wen et al. (2024) demonstrated that *Clonorchis sinensis* EV-delivered csi-miR-96-5p targets PTEN to inhibit ferroptosis in cholangiocarcinoma cells, thereby promoting tumor proliferation and migration (Wen et al., 2024). This observation establishes a direct molecular connection between parasite EVs and carcinogenesis, a finding of considerable clinical significance in regions where liver fluke infections are endemic.

Beyond miRNA-mediated mechanisms, the involvement of EVs in drug resistance has attracted increasing attention. Douanne et al. (2022) provided direct evidence that *Leishmania* parasites exchange drug-resistance genes through EVs, with recipient parasites displaying enhanced growth and improved control of oxidative stress (Douanne et al., 2022). Extending this line of investigation, Tandoh et al. (2025) reported that dihydroartemisinin exposure is positively correlated with EV abundance in *P. falciparum* cultures, and that EVs derived from resistant parasites carry aggregation-prone peptides, suggesting that the EV export pathway may represent a component of the parasite’s adaptive response to artemisinin pressure (Tandoh et al., 2025). These findings introduce the possibility that therapeutically targeting the EV biogenesis or secretion machinery could disrupt the intercellular transmission of drug-resistance traits.

### 4.3 Translational applications: diagnostics, vaccines, and drug delivery

The translational potential of parasite-derived EVs has emerged as a dominant theme in the latter half of the analysis period, with three application domains attracting particular attention.

In the area of diagnostics, the underlying hypothesis is that parasite-derived EVs carry molecular signatures—including proteins, miRNAs, and other nucleic acids—that are indicative of the pathophysiological state of the parent organism and can be detected in host biofluids. Guo et al. (2022) provided proof-of-concept for this approach by identifying three *Echinococcus multilocularis*-derived proteins—thioredoxin peroxidase 1, transitional endoplasmic reticulum ATPase, and 14-3-3—in serum EVs of infected mice, and demonstrating that these markers enabled the detection of echinococcosis as early as 10 days post-infection (Guo et al., 2022). In a clinically relevant demonstration, Meningher et al. (2017) reported that schistosome-specific miRNAs isolated from serum EVs could detect *Schistosoma* infection in returning travelers with 86% sensitivity and 84% specificity, with miRNA levels declining following praziquantel treatment (Meningher et al., 2017). These studies collectively indicate that EV-based liquid biopsy approaches represent a promising avenue for the early and non-invasive diagnosis of parasitic infections.

In the domain of vaccine development, a series of studies has demonstrated that immunization with parasite-derived EVs can confer partial protection against challenge infection across phylogenetically diverse parasite species. Coakley et al. (2017) showed that mice vaccinated with *Heligmosomoides polygyrus* EVs plus alum generated protective immunity against larval challenge, and that this protection was dependent on the IL-33 pathway (Coakley et al., 2017). In a therapeutically relevant context, Chaiyadet et al. (2019) demonstrated that vaccination of hamsters with *Opisthorchis viverrini* EVs and recombinant EV-derived tetraspanins induced antibodies that blocked EV uptake by cholangiocytes and significantly reduced adult fluke recovery after challenge infection (Chaiyadet et al., 2019). More recently, Gao et al. (2022) reported that immunization with *Trichinella spiralis* muscle larvae-derived EVs resulted in 23.4% reduction in adult worms and 43.7% reduction in muscle larvae after challenge, accompanied by a mixed Th1/Th2 immune response (Gao et al., 2022). The convergence of these findings across parasites with distinct tissue tropisms and life histories suggests that the immunogenicity of parasite EVs is a broadly conserved feature that may be systematically exploitable for vaccine design.

For drug delivery applications, the observation that parasite-derived EVs are efficiently internalized by their cognate host cells has motivated the exploration of these vesicles as natural nanocarriers. Borgheti-Cardoso et al. (2020) demonstrated that EVs derived from *P. falciparum*-infected red blood cells could be loaded with the antimalarial drugs atovaquone and tafenoquine, and that such drug-loaded EVs inhibited *in vitro P. falciparum* growth more efficiently than equivalent concentrations of free drug (Borgheti-Cardoso et al., 2020). This study provided an initial proof-of-concept for exploiting the intrinsic tropism of parasite EVs to enhance the delivery and pharmacological efficacy of existing antiparasitic compounds. Such a strategy may prove particularly valuable for intracellular parasites that are naturally protected from many chemotherapeutic agents by host cell membranes.

### 4.4 Methodological challenges and standardization

Despite the considerable progress documented by this analysis, several persistent methodological challenges confront the field. The recent and ongoing citation bursts for the MISEV2018 and MISEV2023 guidelines, as well as the helminth EV-specific community roadmap (White et al., 2023), underscore the recognized need for greater standardization in EV isolation, purification, and characterization protocols. The dual-map overlay analysis suggests a structural basis for this challenge: the biological materials under investigation—particularly EVs released by multicellular helminths with complex life cycles—present technical hurdles that are not adequately addressed by protocols originally optimized for mammalian cell culture systems.

The tension between purity and yield in EV isolation is a particularly salient concern in parasitology, where starting material is frequently limiting. Rigorous characterization of distinct EV subpopulations—as exemplified by comparative analyses of 2K, 10K, and 110K EVs from *Echinococcus granulosus*, which have revealed differential protein cargoes, size distributions, and uptake efficiencies by host cells (Wu et al., 2021; Yang et al., 2021)—is essential to ensure cross-study comparability. Furthermore, the question of which molecular markers are appropriate for identifying and classifying parasite-derived EVs remains unresolved, given that canonical mammalian EV markers such as CD63 and CD81 may be absent or functionally divergent in certain parasite lineages.

### 4.5 Limitations and future directions

Several limitations inherent to bibliometric methodology should be acknowledged. Bibliometric analyses are, by their nature, retrospective and reflect the state of the literature at a specific point in time; as such, the most recent emerging trends may not yet be fully captured. Furthermore, while CiteSpace offers a robust suite of algorithms for network analysis and visualization, the interpretation of clusters and thematic labels remains partially dependent on the analytical parameters selected. Nevertheless, the high modularity and silhouette scores obtained in this study support the reliability of the identified community structures.

Looking forward, the trajectory of the field may be projected along three intersecting fronts. Basic research will continue to dissect the molecular logic of EV-mediated host–parasite communication, with increasing emphasis on *in vivo* validation of mechanisms initially identified *in vitro* and on the deployment of single-vesicle and single-cell technologies. In parallel, a growing translational pipeline is expected to focus on the development of EV-based, field-deployable diagnostic tools for neglected tropical diseases and malaria, the engineering of parasite-derived EVs for prophylactic and therapeutic vaccination, and the exploitation of EV biology for the identification of novel drug targets—for instance, through pharmacological disruption of EV-mediated transfer of drug-resistance genes. Importantly, the sustained citation bursts of methodological and standardization publications extending into 2025 indicate that as these translational initiatives advance, ensuring the rigor and reproducibility of EV research will remain a central, community-wide priority.

## 5 Conclusion

This bibliometric analysis of 365 original research articles published between 2015 and 2025 provides a systematic synthesis of the field of parasite-derived extracellular vesicles. The study reveals that the field has undergone a fundamental transition over the past decade, shifting from predominantly descriptive characterization of EV morphology and cargo toward mechanistic dissection of host–parasite crosstalk and, increasingly, toward translational applications. The conceptualization of parasite EVs has been correspondingly reframed: initially regarded as passive carriers of virulence factors, they are now recognized as central mediators of a complex bidirectional communication network that integrates protein delivery, RNA-mediated cross-kingdom regulation, and immune microenvironment reprogramming. The collaborative network analysis further reveals a structural tension between globally distributed but loosely connected research efforts, highlighting the need for more deeply integrated international partnerships. As methodological standardization advances and single-vesicle technologies mature, the coming decade holds considerable promise for translating the fundamental biology of parasite EVs into tangible tools for the diagnosis, prevention, and treatment of parasitic diseases.

## Data Availability

The raw data supporting the conclusions of this article will be made available by the authors, without undue reservation.
